# Trinculo: Bayesian and frequentist multinomial logistic regression for genome-wide association studies of multi-category phenotypes

**DOI:** 10.1093/bioinformatics/btw075

**Published:** 2016-02-11

**Authors:** Luke Jostins, Gilean McVean

**Affiliations:** ^1^Wellcome Trust Centre for Human Genetics, University of Oxford, Roosevelt Drive, OX3 7BN, UK; ^2^Christ Church, University of Oxford, St Aldates, Oxford OX1 1DP, UK

## Abstract

**Motivation**: For many classes of disease the same genetic risk variants underly many related phenotypes or disease subtypes. Multinomial logistic regression provides an attractive framework to analyze multi-category phenotypes, and explore the genetic relationships between these phenotype categories. We introduce Trinculo, a program that implements a wide range of multinomial analyses in a single fast package that is designed to be easy to use by users of standard genome-wide association study software.

**Availability and implementation**: An open source C implementation, with code and binaries for Linux and Mac OSX, is available for download at http://sourceforge.net/projects/trinculo

**Supplementary information:** Supplementary data are available at *Bioinformatics* online.

**Contact**: lj4@well.ox.ac.uk

## 1 Introduction

Of the many associations discovered by genome-wide association studies, a large number are shared across multiple traits (Parkes *et al.*, 2013), and many more predict different subtypes of the same disease (Taylor *et al.*, 2011). A significant challenge for researchers is finding statistical techniques that leverage this genetic sharing to increase power and discover new biology.

Multinomial logistic regression provides a powerful and flexible framework to carry out association analyses across multiple traits. The frequentist version has been used in studies of disease subphenotypes (Morris, 2010) and in cross-disorder association studies (Smoller *et al.*, 2013). Bayesian extensions have been used to select between different models of genetic sharing across multiple traits (Rockett *et al.*, 2014).

However, these studies have fitted the models in an *ad-hoc* manner, using inefficient R or STATA packages. The lack of a single, flexible tool for multinomial logistic regression has made using these methods difficult for most users, especially when compared to the availability of fast, user-friendly tools for binary logistic regression such as PLINK (Purcell *et al.*, 2007). To address this,we provide a software package that implements a wide range of multi-category logistic analyses in a single efficient and user-friendly program. 

## 2 Functionality

### 2.1 User interface

*Trinculo* uses a command-line interface that is designed to be familiar to users of standard human genetics tools. It uses a PLINK-style format to enter commands and specify input and output files, and reads data in standard formats, including binary PLINK and dosage formats for genotypes and standard text formats for phenotypes and covariates. Sample IDs are automatically matched across different input files, so the user can combine multiple sources of information. Documentation and detailed examples are included with the software.

### 2.2 Use modes

*Trinculo* can carry out a wide range of common multi-category analyses, including:

#### 2.2.1 Frequentist multinomial logistic regression

Calculates a combined (omnibus) *P*-value of association for each variant across all categories using a likelihood ratio test.

#### 2.2.2 Bayesian multinomial logistic regression

Calculates a single Bayes factor for each variant that summarizes the evidence of association across all categories. Users can specify a prior covariance on effect sizes, an independent-effects prior (default) or an empirical prior calculated across all variants.

#### 2.2.3 Bayesian model selection

Generates a marginal likelihood for each possible sharing model, where a sharing model specifies which categories the variant is and is not associated with. The module can calculate Bayes factors in favour of, or against,a variant being shared across categories or uniquely associated to one (see use case below), or posteriors on particular sharing models (if provided with priors on models).

#### 2.2.4 Multi-category simulation

Efficiently simulates genotypes from a multinomial model under ascertainment for given sample sizes and allele frequency. This allows the user to undertake power calculations for the above analyses.

All of these modes can include principal components (to control for population stratification) or other covariates, and can include other SNPs as covariates to test for independent effects or carry out stepwise regression. More details on these use modes, and technical details on their implementation, can be found in Supplementary Materials.

### 2.3 Implementation and speed

*Trinculo* is written in C and is supported on linux and Mac OS X. Models are fitted using Newton’s method, which, after optimization of the second derivative calculation, we find to be much faster than the BFGS method used by other implementations. Like other genetic association software, Trinculo also lends itself readily to parallelization, either by splitting the data up into chunks and running each chunk on a separate core, or through inbuilt multithreading capacity in the software itself.

*Trinculo* can carry out an omnibus frequentist multinomial association scan for a reasonably sized genome-wide association study (1 M SNPs, 4000 cases spread evenly across two categories, plus 2000 controls, with five principal components) on a laptop (1.7 GHz Intel Core i7) in under 10 h. A very large study (100 000 samples across five categories with 50 000 controls) would take 16 h on 24 cores. The fastest R implementation, NNET (Venables and Ripley, 2002),would take 48 h and 5.8 days for the same analyses, respectively. The python implementation statsmodels (Seabold and Perktold, 2010) would take 24 and 31 h, respectively.

## 3 Example use: analysis of inflammatory bowel disease data

**Fig. 1.**
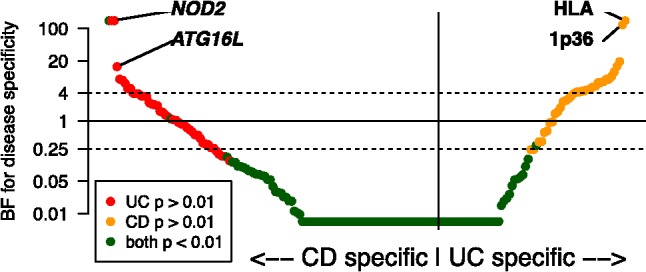
Phenotype specificity Bayes factors for the 193 IBD risk variants. Dots to the left and right of vertical line show stronger evidence of CD and UC specificity, respectively. Colors show classification by *P*-value (a single-disease frequentist association test using binomial logistic regression), dashed lines mark low-certainty assignments (1/4<BF<4). BFs capped at 200 and 1/200 for visibility

We applied the Bayesian model selection mode, using an empirical prior, to data from 193 inflammatory bowel disease risk variants (Jostins *et al.*, 2012). The data came from two IBD phenotypes: Crohn’s disease (CD) and ulcerative colitis (UC),with 17 379 CD cases,13 458 UC cases and 22 442 controls. The disease specific Bayes factors (i.e. the ratio of marginal likelihoods for a model where the variant is only associated with one phenotype and for a model where it is associated with both) for each variant are shown in Figure 1, with the variants with the strongest evidence of phenotype specificity highlighted. We used an empirical prior that estimated the correlation in effect size between the two diseases (estimated as ρ = 0.739).

## 4 Discussion

*Trinculo* is a fast, flexible and easy-to-use tool for multi-category genetic association studies. By providing a wide range of use options, it allows the user to tailor their analysis to their data and experimental design. For instance, if the user wishes to carry out model selection at a risk variant, but wishes to account for the effect of a second risk variant in linkage disquilibrium, then *Trinculo*’s conditional regression option will handle this automatically. Other use cases not discussed here, such a multinomial fine-mapping or ordinal logistic regression, are also included in the software. We hope that these features will allow researchers to use multinomial logistic regression to answer their own biological questions as easily as they currently use binary logistic regression.

## Supplementary Material

Supplementary Data
