# Supplementary material for: Trinculo: Bayesian and frequentist multinomial logistic regression for genome-wide association studies of multi-category phenotypes
Source: Bioinformatics. 2016 Feb 11;32(12):1898–900. doi: 10.1093/bioinformatics/btw075 (PMC4908321; doi:10.1093/bioinformatics/btw075)
Supplement: Supplementary Data [file supp_32_12_1898__index.html]

Trinculo: Bayesian and frequentist multinomial logistic regression for genome-wide association studies of multi-category phenotypes — Trinculo: Bayesian and frequentist multinomial logistic regression for genome-wide association studies of multi-category phenotypes — Supplementary Data 

# Trinculo: Bayesian and frequentist multinomial logistic regression for genome-wide association studies of multi-category phenotypes

## Supplementary Data

files

- Supplementary Data - pdf file
