# Supplementary material for: Trinculo: Bayesian and frequentist multinomial logistic regression for genome-wide association studies of multi-category phenotypes
Source: Bioinformatics. 2016 Feb 11;32(12):1898–900. doi: 10.1093/bioinformatics/btw075 (PMC4908321; doi:10.1093/bioinformatics/btw075)
Supplement: Supplementary Data [file supp_btw075_SupplementaryMethods.pdf]

Supplementary methods for Trinculo: Bayesian and frequentist multinomial logistic regression for genome-wide association studies of multi-category phenotypes

Luke Jostins and Gilean McVean

1 General description of methods

This section gives a text description of the multinomial logistic association framework for genetic association studies, and describes the various tests that *Trinculo* carries out in this framework. Technical details of these methods are given in Section 2.

1.1 Basic description of multinomial logistic regression

When using binomial logistic regression in genetic association studies, we assume that each individual i is either a case (in which case we write $y_i = 1$) or a control ($y_i = 0$). We further assume that the probability of an individual being a case is related to their genotype at some locus $g_i \in (0, 1, 2)$ by the logit link function, given by

$$Pr(y_i = 1|g_i, \beta_0, \beta_1) = \frac{\exp(\beta_0 + \beta_1 g_i)}{1 + \exp(\beta_0 + \beta_1 g_i)}, \quad (1)$$

where β_0 is an intercept and β_1 is an effect size (log odds ratio). We use logistic regression to make conclusions about β_1: what its value is, what the confidence interval is on this estimate, whether this value is larger than zero, etc.

Multinomial logistic regression is an extension of this approach, where instead of each individual being either a case or a control, they are instead either a control ($y_i = 0$) or one of K different case phenotypes $y_i = k$, where $k \in (1, ..., K)$. The probability of being in any one of these categories is given by the multinomial logit link, given by

$$Pr(y_i = k|g_i, \boldsymbol{\beta}_0, \boldsymbol{\beta}_1) = \frac{\exp(\beta_{0k} + \beta_{1k} g_i)}{1 + \sum_{s=1}^{K} \exp(\beta_{0s} + \beta_{1s} g_i)}. \quad (2)$$

In this case there is an intercept β_{0k} and an effect size β_{1k} for each phenotype k. We are no longer learning a single intercept and effect size, but instead a vector of intercepts $\boldsymbol{\beta}_0$ and a vector of effect sizes $\boldsymbol{\beta}_1$. In practice, we generalize this to an arbitrary number of predictors (multiple variants, principal components, other confounders, etc), each with its own vector of effect sizes. We are usually interested in learning things about the components of $\boldsymbol{\beta}_1$: is this vector different from the zero vector (i.e. does this variant effect any phenotypes), which values are significantly larger than zero (i.e. which phenotypes share this genetic variant), how large are they (i.e. how strong an effect does the variant have on each phenotype), etc.

There are two different approaches to learning about $\boldsymbol{\beta}_1$: the frequentist approach, in which we calculate a maximum likelihood estimate of the parameter vector and calculate p-values based on the maximum likelihood, and the Bayesian approach, where we place a prior on the $\boldsymbol{\beta}_1$ and calculate its posterior distribution, and use this to generate Bayes factors based on the marginal likelihood. The frequentist approach is fast and requires few (explicit) assumptions, whereas the Bayesian approach is flexible and allows us to make a broader range of inferences.

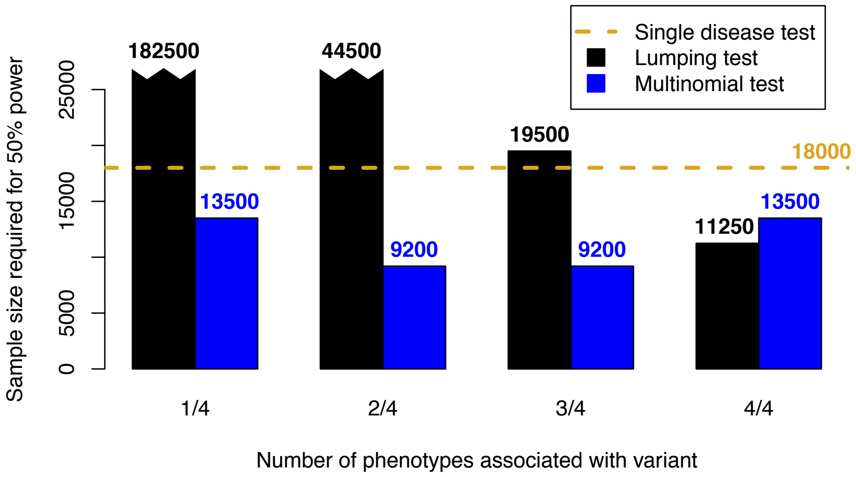

Figure S1: Total sample size (split evenly between phenotypes and controls) required for 50% power to detect association at $p < 5 \times 10^-8$ for an allele with $OR = 1.2$ and minor allele frequency $f = 0.5$ associated with between 1 and 4 of 4 phenotypes.

1.2 Frequentist tests

Fitting the frequentist model by maximum likelihood

We fit the maximum likelihood model using Newton's method (based on first and second derivatives given in the Technical Details section below). This calculates a point estimate for all parameter values (the maximum likelihood estimate, or MLE), as well as standard errors and confidence intervals around the MLE using the Fisher information (the inverse of the second derivative matrix, given in Technical Details).

Omnibus testing using likelihood ratio tests (LRTs)

A typical use of multinomial logistic regression is to improve power of a genome-wide association test by combining cases across multiple phenotypes, and testing whether there is any evidence for an effect of each variant on any of the phenotypes. This works by fitting a full model (including intercepts, genotypic effect sizes and effect sizes for confounders for all phenotypes) and a null model (including only intercepts and effect sizes for confounders, with a null effect of the variant on all phenotypes), and carrying out a likelihood ratio test to see if the full model is a significant improvement over the null model. The omnibus test can gain power by including evidence over multiple associated phenotypes, but also retains most of its power (compared to a single-phenotype test) even if the variant is associated with only one phenotype.

An alternative method is the so-called "lumping" test, in which all cases are treated as a single case set regardless of their specific disease, and binomial logistic regression is carried out on this "lumped" case set. If the variant under consideration is associated with all the diseases in the "lump" with the same effect size this method can be very powerful. However, if the effect sizes are different, or the variant is associated with some phenotypes but not others, this can rapidly dilute the power of the test.

Figure S1 shows an example of the power of the multinomial omnibus test on simulated data. This method improves on the power of the lumping test and the single-disease association test in all cases, except the case of the variant being associated with all phenotypes (where it gives lower but comparable power to the lumping test).

Frequentist single-disease tests using Wald statistics and LRTs

As well as carrying out omnibus tests ("is this variant significantly associated with any of these phenotypes?"), we may also wish to carry out significance tests for each individual phenotype ("is this variant significantly associated with this one phenotype?"). Obviously, these single-phenotype tests can be carried out using ordinary binomial logistic regression, but we can also carry out single-disease tests in the context of frequentist multinomial logistic regression.

Wald tests are a simple way of testing the association for each individual disease by generating a Z-score statistic for each phenotype at that locus, equal to the log-odds ratio divided by the standard error. The per-disease likelihood ratio test is a more involved test, which involves fitting a full model (where the variant is associated with all phenotypes) and a reduced model (in which the variant is associated with all phenotypes other than the phenotype under consideration), and carries out a likelihood-ratio test to see if the former model is a significant improvement over the latter. The second test is significantly slower, and is included more for completeness than for any obvious utility, and in practice gives answers that are very similar to the Wald test.

Note that these tests do not test for evidence in favour of disease specificity; even if only one phenotype is significant, it doesn't follow that the association is specific to this phenotype, as the tests may just lack power for the other phenotypes. Making these sorts of inferences will be discussed in the Bayesian Model Selection section below.

1.3 Bayesian tests

Fitting the Bayesian model by maximum posterior

For the Bayesian analyses we specify a multivariate normal prior on effect sizes by setting a prior covariance (or correlation) matrix. This prior can encode information about the relationship between the effect sizes of the diseases (as described in more detail below, and in Technical Details), and also allows us to generate marginal likelihoods and posteriors that allow us to make a more flexible range of inferences.

During Bayesian model fitting, we estimate and report parameter values that maximise the posterior density (the maximum *a posteriori*, or MAP, estimates) using Newton's method, and approximate the posterior distribution as a normal distribution around these MAP estimates, allowing us to report Bayesian standard errors and 95% credible intervals on parameter estimates. We also calculate and report the marginal likelihood for the model (i.e. the likelihood after integrating out uncertainty in the parameter values). Technical details are given in Section 2.

Omnibus testing using Bayes factors

We can calculate a combined, omnibus Bayes factor in a very similar fashion to the frequentist likelihood ratio test, in order to assess evidence in favour of association between a variant and any of the phenotypes under consideration. This is very similar to the LRT, in that it compares the fit of the full and null models, except that it uses the marginal likelihoods (the likelihood after integrating out uncertainty in the parameter values) rather than maximum likelihoods to compare the models. Unlike the LRT, the omnibus Bayes factor can be greater or less than 1, with numbers greater than 1 indicating evidence in favour of association (i.e. the full model fits the data better than the null mode), and less than one (i.e. the null model fits the data better).

Bayesian fine-mapping

The Bayes factors generated from the Bayesian logistic regression can also be used to carry out fine-mapping across multiple phenotypes in order to identify causal variants, using the approach described in Maller et al[1]. Figure S2 shows a simulated example of how the cross-phenotype fine-mapping approach can significantly increase power to fine-mapping causal variants compared to either fine-mapping a single disease, or lumping all cases together across diseases.

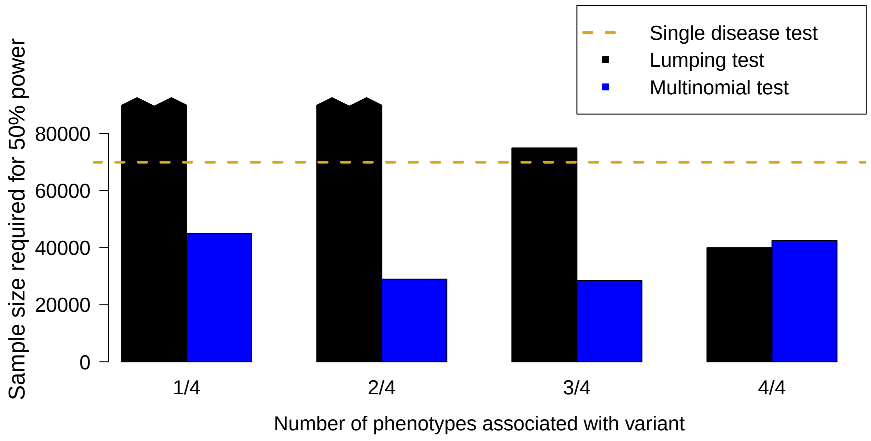

Figure S2: Total sample size required in simulations to have a 50% power to fine-map an association such that the true variant has at least 95% of the posterior. We simulate two variants, the true causal variant and a tag variant with $r^2 = 0.95$ with the true causal variant. Both variants have allele frequency 0.5, the true variant has an odds ratio of 1.2, and samples are equally distributed between the 4 phenotypes and controls. The default prior was used.

Incorporating similarity of diseases using the Bayesian prior

The maximum likelihood based method either treats the two diseases as two separate categories, essentially assuming that the effect sizes are independent (and thus not sharing information across diseases when estimating effect sizes), or treats them as a single category (the "lumping test"), assuming that the effect size is identical.

The Bayesian multinomial regression tests allow a much greater degree of freedom, by explicitly specifying a prior relationship between the effect sizes of the two diseases, in the form of a covariance matrix (or, equivalently, a correlation matrix and a vector of marginal prior variances). If the correlation is close to 1 between two diseases the effect sizes are assumed to be nearly identical (similar to the lumping test), if the correlation is close to zero the effect sizes are assumed to be unrelated (similar to the two-category likelihood-ratio test), and intermediate levels encode differing degrees of similarity. A value of less than zero implies that the two effects often go in different directions.

The correlation relationship between the different diseases can either be user-specified, or if given a large enough set of known associated variants, *Trinculo* can learn it by maximum likelihood (thus making it an empirical prior). Figure S3 shows an example of how accurately the correlation parameter can be inferred as a function of the number of known loci.

Bayesian Model Selection

Many questions we wish to answer in cross-disease association analysis concern what we may call the sharing model for a given variant, i.e. which set of phenotypes is this variant associated with and, crucially, not associated with. Such questions might include "is this variant specifically associated with a single phenotype?", or "is this variant shared by at least two phenotypes?". These questions are complicated by issues of power, as a variant may be truly associated with phenotypes in a way, due to a lack of power to detect it.

The Bayesian framework is well-placed to answer these questions. We can frame them in terms of constraints on the full model, whereby certain parameters are constrained to be zero (as described in Section 2.4), and then integrate out the uncertainty in the remaining parameters to calculate marginal likelihoods, which can in turn be used to calculate Bayes factors (for comparing between two sharing models) or posteriors and credible sets (for a larger number of models). Because uncertainty in parameter values is taken into account, this method is far less sensitive

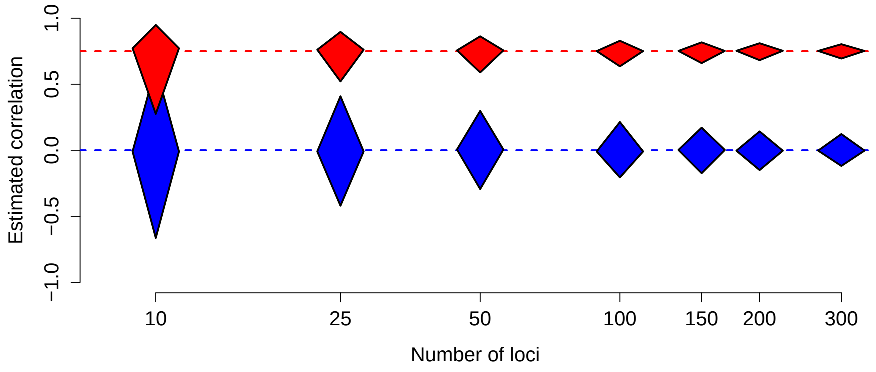

Figure S3: Power to infer the covariance matrix for a given number of loci, using simulated data from 2000 cases of each phenotype, and 2000 controls, with variance in effect size of 0.04. Dashed lines are true values, triangles are 95% range of point estimates.

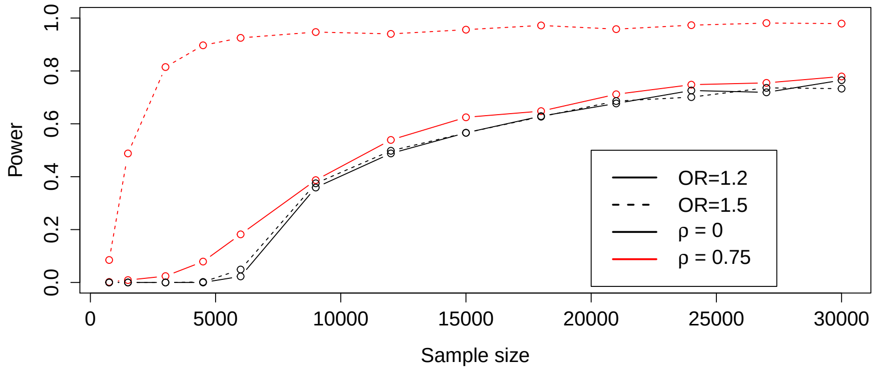

Figure S4: Power to show specificity of association ($BF{>}4$) to one of two phenotypes, given effect size and true correlation. In each simulation the covariance matrix is learned from 200 known loci.

to power issues than frequentist p-value based approaches. Figure S4 shows the power to detect whether an association is specific to one of two phenotypes using this approach.

When you are analysing more than two phenotypes the number of possible sharing models increases rapidly, and the prior you place on these models can have a strong impact on the results. By default, the script included in *Trinculo* assumes a uniform prior across sharing models (i.e. each sharing model is given equal weight). However, this can have unexpected effects, as most of the prior weight is placed on intermediate sharing models where around half of the phenotypes are associated (for instance, for 6 phenotypes there are 20 models where the variant is associated with exactly 3 phenotypes, but only 6 where 1 phenotype is associated, and only 1 model where all six are associated). To get around this, *Trinculo* also supports a prior that is uniform on the number of associated phenotypes (which has the effect of upweighting models that are either widely shared or specific to a single phenotype), as well as user-provided prior files.

2 Technical details

2.1 Notation, likelihood and prior

We generalize the notation given above to include $J+1$ predictors (J variable predictors, plus the intercept), which we will write as a matrix $\mathbf{X}$, with elements x_{ij} (including the intercept, which we will call predictor 0, and write $x_{i0} = 1 \forall i$). We will write $\mathbf{x}_i$ to refer to the vector of predictors for individual i (i.e. the row of the matrix X corresponding to individual i). We write the number of individuals as N.

We will refer to all the effect sizes across all predictors and phenotypes as the effect size matrix $\mathbf{B}$, where $B_{jk} = \beta_{jk}$ is the effect size for predictor j on phenotype k. We will write $\boldsymbol{\beta_k}$ as the vector of effect sizes of all predictors on phenotype k, and $\boldsymbol{\beta_j}$ as the vector of effect sizes of predictor j on all phenotypes.

The probability that an individual is in category k, conditional on their predictors $\mathbf{x_i}$ and parameters $\mathbf{B}$ is given by

$$Pr(y_i = k|\mathbf{B}, \mathbf{x}_i) = p_k(\mathbf{x_i}, B) \tag{3}$$

$$= \begin{cases} \frac{exp(\boldsymbol{\beta}_k^T \mathbf{x_k})}{1+\sum_{s=1}^{K} exp(\boldsymbol{\beta}_s^T \mathbf{x_i})}, & \text{if } y_i > 0. \\ \frac{1}{1+\sum_{s=1}^{K} exp(\boldsymbol{\beta}_s^T \mathbf{x_i})}, & \text{if } y_i = 0. \end{cases} \tag{4}$$

The log likelihood is thus:

$$\ell(\mathbf{B}) = log(Pr(\mathbf{y}|\mathbf{X}, \mathbf{B})) \tag{5}$$

$$= \sum_{i=1}^{N} log(p_{y_i}(\mathbf{x_i}, \mathbf{B})) \tag{6}$$

We set a multivariate normal prior on the effect sizes for each predictor j, such that the log prior is given by

$$Q(\mathbf{B}) = log(Pr(\mathbf{B})) \tag{7}$$

$$= \sum_{j=0}^{J} log(\phi(\boldsymbol{\beta}_j|\boldsymbol{\mu}_j, \boldsymbol{\Sigma}_j)) \tag{8}$$

where $\phi(\cdot)$ is the normal probability density function.

2.2 Choice of priors

In the above section, each predictor j is given its own prior parameters $(\boldsymbol{\mu}_j, \boldsymbol{\Sigma}_j)$. In practice, this would involve setting a very large number of parameters, so we instead just set two different sets of prior parameters, corresponding to the prior on genotypic effect sizes and the prior on nuisance parameters (intercepts and confounders).

The values for the genotypic effects can either be taken as a default value, can be user set, or can be learned from the data (described below). The default prior covariance matrix is a generalisation of the default SNPTest prior, and sets $\Sigma_{ab} = 0.04$ when $a = b$ and $\Sigma_{ab} = 0$ when $a \neq b$. The sign on the effect size is usually arbitrary, as the risk allele is chosen arbitrarily, so for all three cases we set $\boldsymbol{\mu}_j = \mathbf{0}$.

Priors on the effect sizes of intercepts and confounders must be specified as well. Again, we set $\boldsymbol{\mu}_j = \mathbf{0}$ for these values, and $\Sigma_{ab} = 0$ when $a \neq b$ (i.e. effect sizes are distributed around zero and are uncorrelated *a priori*). We also set $\Sigma_{aa} = \sigma$, where σ is single user-specified parameter. Finally, we normalize all confounders to have a variance of 1, to ensure that the effect sizes are

measured on a common scale. We use $\sigma = 1$ by default; investigations in real data suggest that the Bayes factors are largely insensitive to σ, providing it is not set significantly below 1 (data not shown).

2.3 Derivatives of multinomial likelihood and prior

The first and second derivates of the log multinomial likelihood given in section 2.1 are given by

$$U^{\ell}_{ab}(\mathbf{B}) = \frac{\partial \ell(\mathbf{B})}{\partial \beta_{ab}} = \sum_{i=1}^{N} x_{ib}\left[\delta_{y_i,a} - p_a(\mathbf{x_i}, \mathbf{B})\right] \tag{9}$$

and

$$\begin{aligned} V^{\ell}_{abcd}(\mathbf{B}) &= \frac{\partial^2 \ell(\mathbf{B})}{\partial \beta_{ab}\beta_{\ cd}} \\ &= \sum_{i=1}^{N} x_{ib}x_{id}\left[\delta_{a,c}p_a(\mathbf{x_i}, \mathbf{B}) - p_a(\mathbf{x_i}, \mathbf{B})p_c(\mathbf{x_i}, \mathbf{B})\right] \end{aligned} \tag{10}$$

respectively. Here δ_{ab} is the delta function, such that δ_{ab} is equal to 1 if $a = b$, and 0 otherwise.

For the derivatives of the log-prior density, it is easiest to give these in terms of the first and second derivatives of log normal probability density,

$$\boldsymbol{\phi}'_j = \frac{\partial log(\phi(\boldsymbol{\beta}_j|\boldsymbol{\mu}_j, \boldsymbol{\Sigma_j}))}{\partial \beta_j} = -\boldsymbol{\Sigma}_j^{-1}(\boldsymbol{\beta}_j - \boldsymbol{\mu}_j) \tag{11}$$

and

$$\boldsymbol{\phi}''_j = \frac{\partial^2 log(\phi(\boldsymbol{\beta}_j|\boldsymbol{\mu}_j, \boldsymbol{\Sigma_j}))}{\partial \beta_j^2} = -\boldsymbol{\Sigma}_j^{-1}. \tag{12}$$

The first and second derivates of the log prior density are thus

$$U^{Q}_{ab}(\mathbf{B}) = (\boldsymbol{\phi}'_b)_a \tag{13}$$

and

$$V^{Q}_{abcd}(\mathbf{B}) = \frac{\partial^2 Q(\mathbf{B})}{\partial \boldsymbol{\beta}_{l=b}\boldsymbol{\beta}_{l=d}} = \delta_{bd}(\boldsymbol{\phi}''_b)_{ac} \tag{14}$$

respectively.

2.4 Calculating marginal likelihoods and Bayes factors

We often wish to fit constrained models where the effects of particular predictors on particular phenotypes (i.e. particular elements of $\mathbf{B}$) are set to zero. We define a particular model M, such that M specifies the set of constrained predictors and phenotypes, i.e. $\beta_{ab} = 0 \ \forall (a, b) \in M$. We can calculate marginal likelihoods for a given model as

$$Pr(\mathbf{y}|\mathbf{X}, M) = \int_{\beta_{ab}:(a,b)\notin M} e^{\ell(\mathbf{B})+Q(\mathbf{B})} d\mathbf{B}, \tag{15}$$

which we approximate using Laplace's method as

$$\begin{aligned} P(M|\mathbf{X}, \mathbf{y}) \approx (2\pi)^{\frac{J+1}{2}} & \left|V^{\ell}(\hat{\mathbf{B}}_{MAP}) + V^{Q}(\hat{\mathbf{B}}_{MAP})\right|^{\frac{1}{2}} \\ & \times \ \exp\left(\ell(\hat{\mathbf{B}}_{MAP}) + Q(\hat{\mathbf{B}}_{MAP})\right), \end{aligned} \tag{16}$$

where $\hat{\mathbf{B}}_{MAP}$ is the maximum *a posteriori* estimation of $\mathbf{B}$, calculated using Newton's method to maximise $\ell(\mathbf{B}_{MAP}) + Q(\mathbf{B})$, with first and second derivatives given by $U^{\ell}(\mathbf{B}) + U^{Q}(\mathbf{B})$ and $V^{\ell}(\mathbf{B}) + V^{Q}(\mathbf{B})$ respectively.

We can use these marginal likelihoods to calculate Bayes factors between models $BF_{M_1,M_2} = \frac{Pr(\mathbf{y}|\mathbf{X},M_1)}{Pr(\mathbf{y}|\mathbf{X},M_2)}$. For instance, if we set $M_1 = \{(1,1)\}$ (i.e. $\beta_{11} = 0$ and $\beta_{21} \neq 0$) and $M_2 = \emptyset$ (i.e. an unconstrained model with $\beta_{11} \neq 0$ and $\beta_{21} \neq 0$), then BF_{M_1,M_2} would be the evidence in favour of predictor 1 being specifically associated with phenotype 2 but not phenotype 1. Or, if $M_1 = \emptyset$ and $M_2 = \{(1,1),(1,2)\}$ then BF_{M_1,M_2} corresponds to the omnibus evidence in favour of association across both phenotypes. The marginal likelihoods can also be used in combination with a per-model prior to calculate posteriors across models. A python script is included in the *Trinculo* package to convert the marginal likelihood output into various Bayes factors and posteriors.

2.5 Estimating an empirical covariance matrix prior

If we have a large number of loci, and we assume that their effect sizes are all drawn from the same distribution, we can estimate a single underlying covariance matrix $\mathbf{\Sigma}_l = \mathbf{\Sigma}$ across all loci l. For each locus we fit the maximum likelihood model described above and calculate the estimated genotypic effect size vector $\hat{\boldsymbol{\beta}}_l$ and the error covariance matrix $\hat{\mathbf{\Omega}}_l$. We can then model the estimated effect sizes for each locus as being drawn from the distribution $\hat{\boldsymbol{\beta}}_l \sim N(0, \mathbf{\Sigma} + \hat{\mathbf{\Omega}}_\mathbf{l})$, and define a log-likelihood $\ell(\mathbf{\Sigma}) = \sum_l log(\phi(\hat{\boldsymbol{\beta}}_l|0, \mathbf{\Sigma} + \hat{\mathbf{\Omega}}_l))$. We calculate the maximum-likelihood estimate, $\hat{\mathbf{\Sigma}}$, using Newton's method. This estimated covariance matrix can in turn be used as an empirical prior for calculating marginal likelihoods for individual loci.

The first and second derivates of $\ell(\mathbf{\Sigma})$ are given by

$$\frac{\partial \ell(\mathbf{\Sigma})}{\partial \mathbf{\Sigma}} = \sum_{l=1}^{L} \left[\mathbf{W}_l - \mathbf{W}_l \hat{\boldsymbol{\beta}}_l \hat{\boldsymbol{\beta}}_l^T \mathbf{W}_l\right] \circ \left[\mathbf{1} - \frac{\mathbf{I}}{2}\right] \tag{17}$$

and

$$\frac{\partial^2 \ell(\mathbf{\Sigma})}{\partial \mathbf{\Sigma} \partial \Sigma_{ab}} = \sum_{l=1}^{L} \left[-\mathbf{W}_l \mathbf{S}^{ab} \mathbf{W}_l + \mathbf{W}_l (\mathbf{S}^{ab} \mathbf{W}_l \hat{\boldsymbol{\beta}}_l \hat{\boldsymbol{\beta}}_l^T + \hat{\boldsymbol{\beta}}_l \hat{\boldsymbol{\beta}}_l^T \mathbf{W}_l \mathbf{S}^{ab}) \mathbf{W}_l\right] \circ \left[\mathbf{1} - \tfrac{\mathbf{I}}{2}\right] \tag{18}$$

respectively, where $\mathbf{W_l} = (\mathbf{\Sigma} + \hat{\mathbf{\Omega}}_l)^{-1}$, $\mathbf{A} \circ \mathbf{B}$ is the Hadamard (i.e. element-wise) product, $\mathbf{1}$ is a matrix where all elements are equal to 1 and $\mathbf{S}^{ab}$ is a matrix where all elements are equal to zero except $(S^{ab})_{ab} = (S^{ab})_{ba} = 1$.

2.6 Simulation methodology

This simulation scheme is designed to efficiently sample genotype information from multiple phenotypes given confounding and ascertainment bias, even when certain categories are very rare. The aim is to simulate a set of predictors (genotypes and confounders) for each individual $\mathbf{x_i}$, conditional on a phenotype for each individual y_i. We will write $\mathbf{x_i} = \{1, g_i, \mathbf{x}_i^c\}$, where the zeroth predictor is the intercept ($x_{i0} = 1$), the first predictor is g_i, the genotype for this individual, predictors 2 to J are the confounders $\mathbf{x}_i^c$ (giving $J-1$ confounders). The simulation schedule requires the following parameters:

- the number of phenotypes K,
- the number of individuals to sample in each phenotype category N_k,
- a population prevalence of each phenotype π_k,
- the population allele frequency of the risk variant f,

- the number of confounders $J-1$
- the effect of each confounder on each phenotype β_{kj},
- the effect of each confounder on the allele frequency c_j (e.g. from the results of a regression of confounders on dosage in a reference population),
- the effect size of the risk variants on each phenotype, either set directly as β_{k1} or from a provided covariance matrix $\mathbf{\Sigma}$ to draw zero-centered multivariate normal values from.

The intercept values β_{k0} are set to give the given phenotype population frequencies π_k, using the approximate equation

$$\beta_{k0} = log(\pi_k) - log(\pi_0) - 2f\beta_{k1} - 2f(1-f)\beta_{k1}^2 - \sum_{j=2}^{J} \beta_{kj}^2. \tag{19}$$

We calculate the mean value of each confounder in each phenotype using numeric integration, and sample confounders independently. We can then calculate the genotype probabilities conditional on disease status and confounders using

$$Pr(g_i|y_i = k, \mathbf{x_i^c}) = \frac{p_k(\{1, g_i, \mathbf{x_i^c}\}, \mathbf{B})Pr(g|\mathbf{x_i})}{\sum_{g'} p_k(\{1, g', \mathbf{x_i^c}\}, \mathbf{B})Pr(g'|\mathbf{x_i^c})}, \tag{20}$$

where $Pr(g|\mathbf{x^c})$ is the population frequency of genotype g, given an adjustment for confounding between genotype and confounders. We model this confounding as $g_i|\mathbf{x_i^c} \sim Binom(2, f + \mathbf{c}^T\mathbf{x_i^c})$.

References

[1] Maller, J. B. *et al.* (2012). Bayesian refinement of association signals for 14 loci in 3 common diseases. *Nat. Genet.*, **44**(12), 1294–1301.
